# PREVALENCE AND THE ASSOCIATED FACTORS OF MULTIMORBIDITY AMONG OLDER NEPALIS IN WESTERN NEPAL

**DOI:** 10.1093/geroni/igae098.1182

**Published:** 2024-12-31

**Authors:** Lirisha Tuladhar, Krishna Sapkota, Bharat Kafle, Preeti Bhattarai, Pratik Bhattarai, Aman Shrestha

**Affiliations:** Miami University, Oxford, Ohio, United States; Miami University, Oxford, Ohio, United States; Karnali Academy of Health Sciences, Jumla, Karnali, Nepal; Karnali Academy of Health Sciences, Jumla, Karnali, Nepal; Oxford University Clinical Research Unit Nepal, Lalitpur, Bagmati, Nepal; University of Maryland School of Medicine, Baltimore, Maryland, United States

## Abstract

Multimorbidity, the concurrent presence of multiple chronic diseases, is a global public health issue, with higher prevalence rates observed among older age groups and individuals of lower socioeconomic status. This study aims to determine the prevalence of morbidity levels and the associated factors across all three ecological regions of Western Nepal. Utilizing a community-based cross-sectional design, 612 participants were interviewed face-to-face. Levels of morbidity were grouped as “no morbidity,” “single disease,” and “multiple diseases.” Ordinal logistic regression examined the association between levels of morbidities and various sociodemographic, economic, and health behavior factors. Probabilities models were cumulated over the higher-ordered levels of morbidity. Among sociodemographic variables, participants of urban residents (OR=1.68, CI: 1.16—2.44), of age 70 years and above (OR=1.55, CI: 1.10—2.17), females (OR=2.43, CI:1.61—3.66) and with disability (OR=1.58, CI:1.14—2.18) were associated with increased odds of being in a higher category (increasing morbidity) rather than lower category than their respective counterparts after adjusting for the covariates. Economic factors did not establish significant relationships with morbidity levels. Among health behavior factors, those who consumed alcohol had 41% decreased odds of higher morbidity compared to non-consumers (OR=0.59 CI:0.37—0.92). This study identified significant associations between multimorbidity and specific demographic groups in urban areas, notably older females and individuals with disabilities. It highlights the necessity of providing tailored behavior change information and implementing screening programs to reduce the prevalence of health issues in these populations.

